# Highly Reliable Ovonic Threshold Switch with TiN/GeTe/TiN Structure

**DOI:** 10.3390/ma16052066

**Published:** 2023-03-02

**Authors:** Dongjun Seong, Su Yeon Lee, Hyun Kyu Seo, Jong-Woo Kim, Minsoo Park, Min Kyu Yang

**Affiliations:** 1 Artificial Intelligence Convergence Research Lab, Sahmyook University, Seoul 01795, Republic of Korea; 2 Smith College of Liberal Arts, Sahmyook University, Seoul 01795, Republic of Korea

**Keywords:** ovonic threshold switching, crossbar array, 1S1R, ReRAM

## Abstract

A new architecture has become necessary owing to the power consumption and latency problems of the von Neumann architecture. A neuromorphic memory system is a promising candidate for the new system as it has the potential to process large amounts of digital information. A crossbar array (CA), which consists of a selector and a resistor, is the basic building block for the new system. Despite the excellent prospects of crossbar arrays, the biggest obstacle for them is sneak current, which can cause a misreading between the adjacent memory cells, thus resulting in a misoperation in the arrays. The chalcogenide-based ovonic threshold switch (OTS) is a powerful selector with highly nonlinear *I–V* characteristics that can be used to address the sneak current problem. In this study, we evaluated the electrical characteristics of an OTS with a TiN/GeTe/TiN structure. This device shows nonlinear DC *I–V* characteristics, an excellent endurance of up to 10^9^ in the burst read measurement, and a stable threshold voltage below 15 mV/dec. In addition, at temperatures below 300 °C, the device exhibits good thermal stability and retains an amorphous structure, which is a strong indication of the aforementioned electrical characteristics.

## 1. Introduction

The success of today’s computing systems is due to the von Neumann architecture, whose design involves moving data back and forth between the processor and the memory. There are two major reasons for the success of this architecture. The first one is its Turing completeness, which means that, given a sufficient amount of memory and time, it can complete any mathematical task. The second one is its scalability, which makes it possible to expand the memory. As the amount of data increases, the computation speed also increases. There is no need to modify the architecture or the associated programming model [1,2,3,4,5,6,7,8,9,10,11,12,13,14,15]. However, this data transfer system accounts for a large part of the power consumed. Even though the next-generation computer systems can perform exascale (10^18^) calculations per second, enabling them to deal with complex data, they will consume about 30 MW of power when using the von Neumann architecture. Furthermore, the von Neumann system suffers from latency owing to the huge amount of data transfer between the separate memory unit and logic unit [16,17]. This problem resulted in the development of a neuromorphic computing system, which is inspired by the biological neural system and has a much lower power consumption than that of conventional processors. This new computing system has the potential to process large amounts of digital information and can be used for natural language processing, driving automation, and big data analysis.

A crossbar array (CA) is the basic building block of the neuromorphic system; it is composed of a memory and a selector. The memory acts as a synaptic weight for storing information and processing of input signals. Among the candidates for the memory, resistive random-access memory (RRAM) is the most promising one because of its high capacity, multilevel programming, high speed, and scalability down to the 4F^2^ design rule. The role of the selector is to prevent sneak current through unchosen cells, which leads to disruption in data reading and programming [18,19,20,21,22,23,24,25]. The chalcogenide-based ovonic threshold switch (OTS) is currently the best device for the selector. The OTS has a fast switching speed, bidirectional rectifying characteristics, and CMOS compatibility [26,27,28,29,30,31,32]. Furthermore, it uses amorphous materials, enabling it to achieve its fast switching speed and nonlinearity in *I–V* characteristics [33,34,35,36,37,38].

In this study, we investigated an OTS device, namely, GeTe (GT), which has shown excellent thermal stability and electrical endurance characteristic.

## 2. Materials and Methods

**Figure 1:**
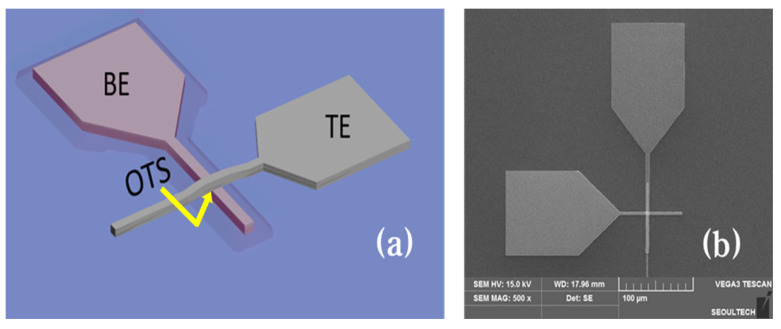
(**a**) Schematic diagram of the BE TiN/OTS (GT)/TE TiN structure and (**b**) SEM image of the same device.

***Device Fabrication*:**Figure 1a shows a schematic diagram of the top electrode (TE) TiN/OTS (GT)/bottom electrode (BE) TiN structure. A heavily doped P-type Si wafer (*ρ* < 0.01 Ωcm) and a thermally oxidized SiO_2_ wafer with a dimension of 300 nm were used as the substrates. The BE was patterned using an inductively coupled plasma-reactive ion etching method after depositing the TiN with a physical vapor deposition system.

A radio frequency (RF) sputtering system with a Ge-Te (1:1) target was used to deposit the GT layers. The basal vacuum pressure in the chamber was set to less than 1.0 × 10^−6^ Torr, and the working pressure of the Ar gas (purity of 99.999%) and RF power were maintained at 2.0 × 10^−3^ Torr and 50 W, respectively, during sputtering. Before the deposition, pre-sputtering was performed for 30 min to eliminate any contaminants on the target. After the deposition of the GT layers, TE TiN was deposited and patterned using the lift-off method. Figure 1b shows a top-view image of the device.

***Materials Analysis*:** The samples for material analysis were prepared simultaneously for the GT device on the SiO_2_/Si substrate. The sample for the high-resolution transmission electron microscopy (HR-TEM) analysis was prepared by a focused ion beam operation (FIB (Leeuwarden, The Netherlands), Helios NanoLab™ (Sarasota, FL, USA), FEI (Hillsboro, OR, USA)). HR-TEM (Tecnai G2 F30 S-TWIN, FEI) analysis was then performed to obtain a cross-sectional view of the Si/SiO_2_/TiN/GeT/TiN stacked device.

***Electrical Measurements*:** The electrical characteristics of the device were measured using an HP4145B semiconductor parameter analyzer (SPA) in the DC *I–V* sweep mode. The temperature was controlled by a hot-stage microscope using a temperature controller. Pulse-based electrical measurements were conducted using the HP4145B, an arbitrary function generator (Agilent 81150 A, Santa Clara, CA, USA), an oscilloscope (MSOX3024T, Tektronix, Beaverton, OR, USA), and an electromechanical RF electrical circuit switch box. Throughout the measurement process, the voltage was biased to the TiN TE, while the TiN BE was electrically grounded. The resistance (or current) values of the programming and erasure were verified at 2 V using the SPA. These two types of electrical circuits were alternately used by the electromechanical RF electrical circuit switch boxes. All the electrical measurements were performed using a LabVIEW-based control program.

## 3. Results

**Figure 2:**
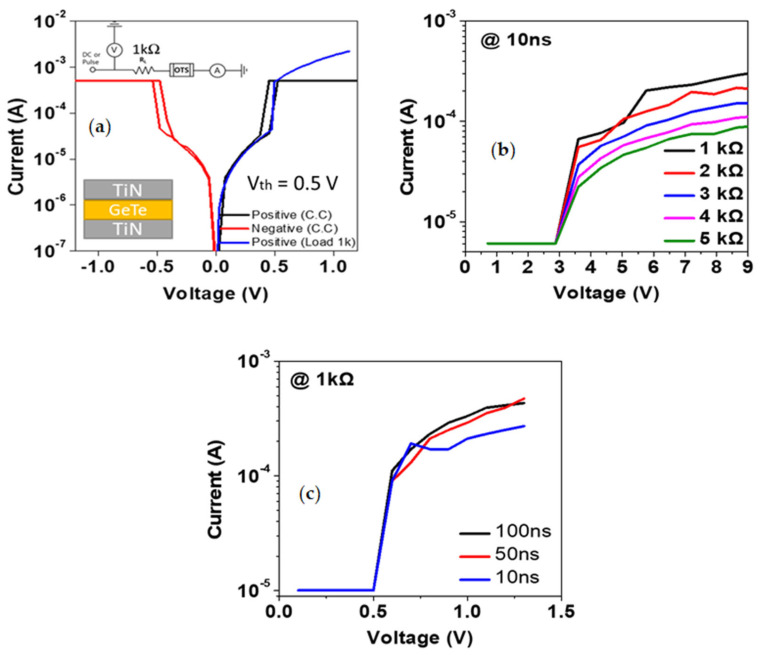
(**a**) DC *I–V* characteristic curves of the BE TiN/OTS (GT)/TE TiN device. The black line is the sweep curve for the positive bias region, and the red line is that for the negative bias region. A current compliance of 500 µA was applied to the device to prevent a permanent breakdown. The blue line is the DC *I–V* characteristic curve under the 1-kΩ external load resistor. (**b**) Pulse-based current voltage characteristics showing forming voltage values of devices with different external load resistances. (**c**) Pulse-based current voltage characteristics of the device with different pulse widths under the 1-kΩ external load resistor.

Figure 2a shows the DC *I–V* characteristic curves of the TiN/OTS (GT)/TiN device, which were measured by applying a DC bias. We set the compliance current to prevent a dielectric breakdown of the device. The black lines in the positive bias region and the red lines in the negative bias region were the results of the measurement under a current compliance of 500 µA. Moreover, the blue line is the characteristic under the bias of an external 1-kΩ load resistor. We found that the threshold voltage (Vth) of this device was under 0.5 V during the forward voltage sweep. At Vth, the OTS device maintained a high resistance state (off state), and the current increased abruptly (on state). During the backward sweep, the on state returned to the off state again.

The OTS device exhibited identical values of Vth. Considering that typical OTS devices generally have different Vth values, our OTS device shows special features and, thus, should be investigated further [28,29]. In the results of the study, Valea, A. et al., it can be confirmed that the Vth is 1.4 V, which is different from the Vth (0.5 V) of our OTS device. In addition, the results of studies by Laguna, C. et al. also show that Vth is different from ours. Figure 2b shows the pulse-based current voltage (PIV) characteristics of the device with different external load resistors. Here, the pulse is ISPP (Incremental step pulse programming), and a method of measuring while continuously increasing the amplitude value in the same pulse width state is applied. During the PIV measurement, a series of pulses with different voltages was applied, and the resistance value of each step was measured using an oscilloscope. To prevent the device from breakdown during AC measurement, we set the external load resistance from 1 kΩ to 5 kΩ to check for breakdown voltage, which did not occur. As the external resistance approaches zero, more current flows through the element. Therefore, the pulse width was changed from 10 ns to 100 ns at a fixed external load of 1 kΩ to determine the minimum pulse width for proper operation of the device, as shown in Figure 2c.

The OTS is not used alone but is connected in series to the RRAM in the CA. The most important role of the OTS is to prevent a sneak current during a read operation. The leakage paths through unselected cells in the CA can lead to an inaccurate output signal and clear identification of the high-resistance state from the low-resistance state of the RRAM cell. Figure 2 shows the nonlinear characteristics of the OTS, which can suppress sneak current and thus improve the read margin of the CA.

**Figure 3:**
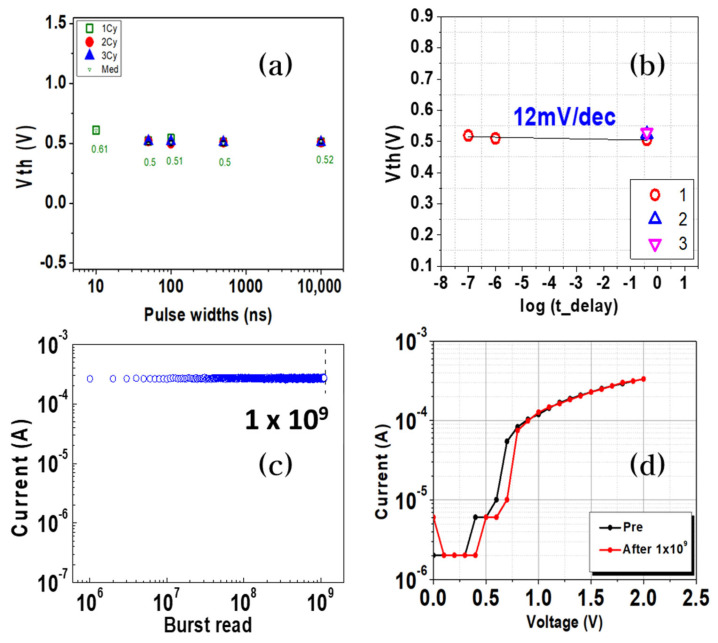
(**a**) Threshold voltage (Vth) endurance with different pulse widths. Even though the pulse widths were changed from 10 ns to 10 us, the threshold voltage maintained almost the same value. (**b**) Vth shift with delay time on a log scale. Vth was retained under 15 mV/dec, which shows stable characteristics. (**c**) Results of the burst read endurance test showing excellent endurance. Read voltage equal to Set voltage (=0.5 V). The burst read scheme differs from the conventional one, which repeats the write/read operation. The resistance of the OTS was determined after 10 to *n* numbers of pulses were applied to the OTS cell. (**d**) Comparison of the PIV results before and after the endurance test. We found robust OTS characteristics, which show good switching characteristics after 10^9^ cycles of the endurance test.

Figure 3a shows the Vth change with different pulse widths. We changed the pulse widths from 10 ns to 10 µs and checked the Vth value of each state, which showed little change. It has excellent stability, and the result has significance for CA applications. Because the OTS is part of the 1 Selector-1 Resistance (1S1R) CA, how the RRAM works is very important. Therefore, the operating voltage of the memristor affects the operation of the selector. The maximum operating voltage of the memristor that the selector can operate is up to 3 V. However, if the voltage is greater than 3 V, breakdown may occur in the initial cycle, so the lower the voltage, the better. The current trend for increasing the memory density is by using multi-bit operation, which is a very effective way to store more information in a single device. For the RRAM, multi-bit resistive switching has been reported in previous studies by setting the current compliance, changing the reset voltage, and modulating the voltage pulse amplitude or height. Among those studies, Alamgir et al. reported the feasibility of the multi-bit operation of the RRAM by modulating the pulse width [39,40,41]. They demonstrated this possibility by changing the pulse width without any overlapping of the resistance levels. If the OTS is serially connected to the RRAM, the crucial role of the OTS is to maintain Vth while voltages of different pulse widths are applied to the cell. Figure 3b shows the Vth shift with delay time.

After the OTS was turned on, the values of Vth were measured at different times on a log scale to check whether the OTS maintained its initial value. The OTS endurance was measured because the reliability of the OTS is a key issue that can affect the performance and accuracy of a large-scale cell array. The burst read endurance was measured, as shown in Figure 3c. In contrast to nonvolatile memory, such as the RRAM, the OTS is a volatile device. The burst read scheme differs from the conventional one, which repeats the write/read operation. After 10 to *n* numbers of pulses were applied to the OTS cell, the OTS resistance was determined. Through this method, we can reduce the read damage using the conventional read scheme. Figure 3d shows a comparison of the PIV results before and after the endurance test. Robust OTS characteristics showed good switching characteristics after 10^9^ cycles of the endurance test. From the results, we can confirm the robust endurance of the OTS cell.

**Figure 4:**
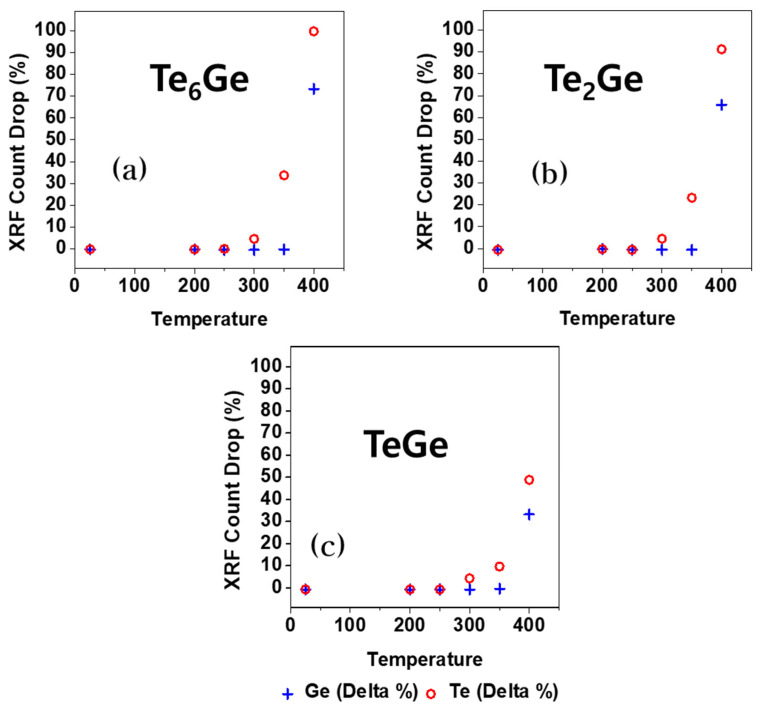
Drop in the XRF count of the OTS device with increasing temperature. (**a**–**c**) are the Te_6_Ge, Te_2_Ge, and TeGe, respectively.

To confirm the thermal stability, we performed an XRF analysis on the different components of the GT samples. A quantitative analysis of the Ge and Te of the compounds was performed every 50 °C after 200 °C. As shown in Figure 4a–c, the quantity of Te dramatically decreases after 350 °C, and the rate of decrease of Te is higher than that of Ge at 400 °C.

**Figure 5:**
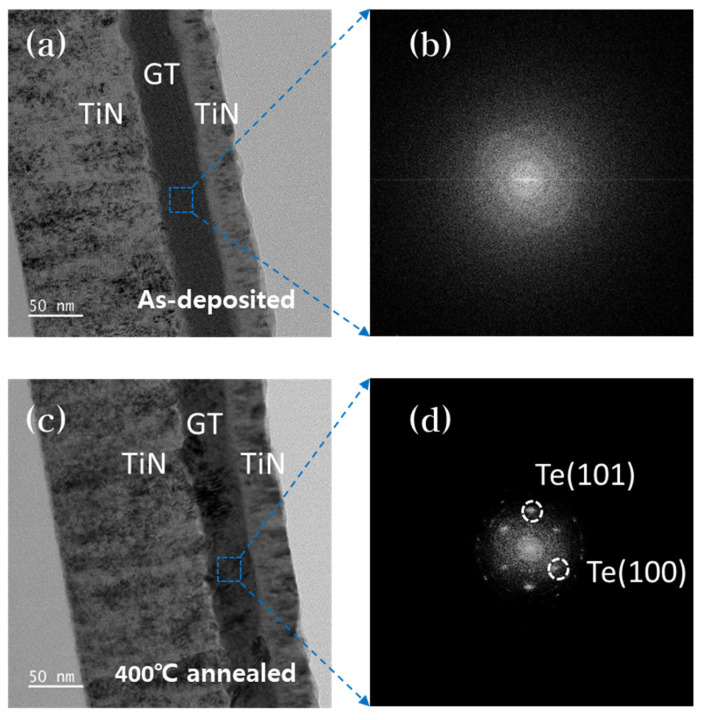
(**a**,**c**) are cross-sectional view before and after annealing GT with TiN/GT/TiN structures obtained by high-resolution transmission electron microscope (HR-TEM) analysis for direct observation of GT thin films. (**b**,**d**) show the FFT results of (**a**,**c**) layers.

We performed an HR-TEM analysis for the direct observation of the GT thin film. Figure 5a shows a cross-sectional view of a TiN/GT/TiN structure before annealing the GT layer. Figure 5c shows a cross-sectional view of the same structure after 400 °C annealed. We analyzed the diffraction pattern in the blue dotted-ray rectangular region visible in the GT layer according to the fast Fourier transform (FFT) method. Figure 5b shows the diffraction pattern in the GT layer before annealing, and Figure 5d shows the pattern after 400 °C annealing. The FFT results of the GT layer before annealing showed amorphous characteristics, as illustrated in Figure 5b, and crystalline Te could be observed in the GT layer after annealing. This indicates that GT loses its amorphous properties at this temperature.

The biggest advantage of the OTS is its highly nonlinear current flow (in the DC *I–V* characteristic), which plays a crucial role in preventing sneak current in the 1S1R cell array structure, as mentioned earlier. In particular, the endurance of the OTS in pulse-based operations must be greater than that of the resistor because the OTS is turned on for every programming and reading event. The amorphous structure of the OTS is responsible for this endurance, which offers electronic trap sites between the conduction band and the Fermi level. Trapping and de-trapping by the external electric field can cause high nonlinearity.

**Figure 6:**
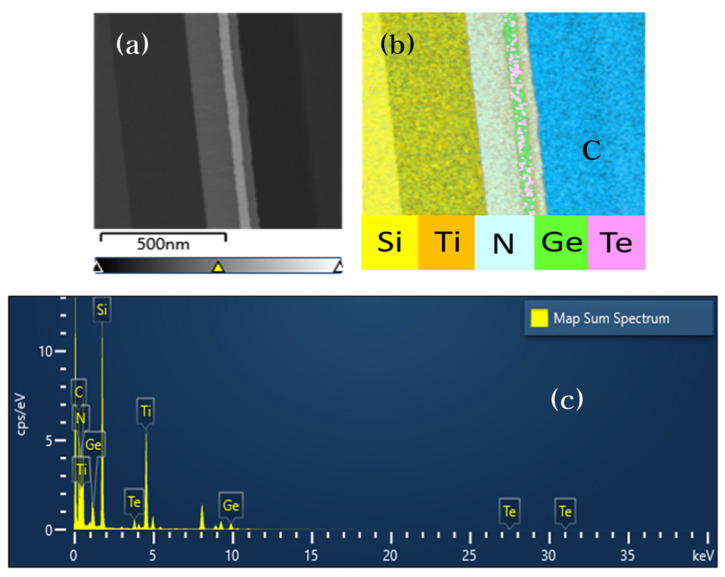
(**a**) Cross-sectional view of the Si/SiO2/TiN/GT/TiN device. The top of the sample carbon layer was deposited for analysis. (**b**) Map of the sample with every element of the sample. (**c**) Map sum spectrum of the device.

To investigate the composition of the device, we performed a transmission electron microscopy (TEM)/energy dispersive X-ray spectroscopy (EDS) analysis on the sample. Figure 6a shows a cross-sectional image of the TiN/GT/TiN device obtained by TEM analysis, and Figure 6b illustrates the results of the EDS analysis of the cross-section of the device. We can confirm that Te and Ge coexist between the bottom and top TiN layers. Figure 6c shows the map sum spectrum of the sample. The elements and atomic ratios of the TiN/GT/TiN device are shown in Table 1.

## 4. Conclusions

In summary, in this study, the electrical characteristics of an OTS with a TiN/GT/TiN structure were investigated. This device shows nonlinear DC *I–V* characteristics and excellent endurance of up to 10^9^ in the burst read measurement. Considering that the OTS is turned on for every programming and reading event, the endurance of the OTS must be greater than that of the resistor, and the device meets this requirement. In addition, the device exhibited a stable threshold voltage below 15 mV/dec. The thermal stability indicates a stable thermal property below 300 °C. The thermal stability above this temperature requires further study. Moreover, the device retains an amorphous structure below 300 °C, which is a strong indication of the aforementioned electrical characteristics.

## Figures and Tables

**Table 1:** Map sum spectrum of the TiN/GT/TiN device.

Element	Weight %	Weight % Sigma	Atomic %
C	31.91	0.29	52.74
N	13.34	0.33	18.90
Si	26.03	0.22	18.40
Ti	19.93	0.17	8.26
Ge	2.83	0.05	0.77
Te	5.96	0.63	0.93
Total	100.00		100.00

## Data Availability

Not applicable.
